# ‘Come and work here!’ a qualitative exploration of local community-led initiatives to recruit and retain health care staff in remote and rural areas of the UK

**DOI:** 10.1177/13558196251318607

**Published:** 2025-03-02

**Authors:** Andrew Maclaren, Louise Locock, Zoë Skea, Lorraine Angell, Jennifer Cleland, Topher Dawson, Alan Denison, Christina Dobson, Rosemary Hollick, Peter Murchie, Diane Skåtun, Verity Watson

**Affiliations:** 1 Former Lecturer, Institute of Applied Health Sciences, 1019 University of Aberdeen, Aberdeen, UK; 2 Professor, Aberdeen Centre for Evaluation, 1019 University of Aberdeen, Aberdeen, UK; 3 Advanced Research Fellow, Aberdeen Centre for Evaluation, 1019 University of Aberdeen, Aberdeen, UK; 4 Public Research Partner, UK; 5 Professor, Medical Education Research & Scholarship Unit, 54761 Nanyang Technological University, Singapore; 6 Public Research Partner, Ross and Cromarty, UK; 7 Professor and Dean of Postgraduate Medicine, 9529 NHS Education Scotland, Aberdeen, UK; 8 Senior Research Associate, Population Health Sciences Institute, 5994 Newcastle University, Newcastle upon Tyne, UK; 9 Senior Clinical Lecturer and Consultant Rheumatologist, Epidemiology, 1019 University of Aberdeen, Aberdeen, UK; 10 Professor, Academic Primary Care, 1019 University of Aberdeen, Aberdeen, UK; 11 Senior Research Fellow, Health Economics Research Unit, 1019 University of Aberdeen, Aberdeen, UK; 12 Professor, Health Economics Research Unit, 1019 University of Aberdeen, Aberdeen, UK

**Keywords:** community, healthcare workforce, qualitative, qualitative methods, recruitment and retention, rural health care

## Abstract

**Objectives:**

The recruitment and retention of health care staff to remote and rural communities is a major challenge. This study explored the experiences of remote and rural communities in trying to attract and retain health care staff and their families in the UK.

**Methods:**

Qualitative case studies in five remote and rural communities, two in England and three in Scotland. We conducted interviews with 22 participants across the five sites, including community members actively involved in recruitment and retention, health care professionals, and their family members. Fieldwork combined remote and in-person data collection. We used thematic analysis across cases drawing on asset-based community development as our theoretical framework.

**Results:**

Communities undertook various activities such as making promotional videos, social media campaigns, getting involved on interview panels, and informal social integration efforts. They drew on a range of local assets to encourage health care staff to come to the area, including showcasing local landscapes, outdoor activities, a safe, welcoming community for children and families, and good quality of life. They also drew on the skills of local people with backgrounds in marketing, design, communications and photography or film-making. The absence of some key assets posed challenges, particularly lack of housing, schooling provision, employment opportunities for other family members, and cultural activities. Community-led initiatives were often prompted by local dissatisfaction with health organisations’ efforts to recruit health care staff, and a wish to exercise some control over recruitment initiatives. Activities were commonly driven by a small number of individuals. While this worked well in some communities, the burden of responsibility could be substantial. This also sometimes led to tension within communities. Retention efforts commonly relied on informal networks of key individuals who intentionally forged social links for incoming families.

**Conclusions:**

There is a key role for communities to play in recruitment and retention in remote and rural regions. There is an opportunity to actively engage community members in collaboratively crafting a campaign that highlights the area’s key attractions while being mindful of limitations on reliance on an asset-based approach. Retention is a neglected topic, relying on key individuals going out of their way to help newcomers integrate. The formation of a community stakeholder group could help formalise this and reduce reliance on the goodwill and energy of individuals.

## Introduction

Recruitment and retention of health care workers is an important challenge for health and social care globally, particular in rural and remote areas,^1–3^ widening inequalities in health care access and outcomes.^4,5^ Robust evidence of remote and rural health care workforce interventions remains limited.^6,7^ The best predictor of career intention to work in these settings is being born or brought up in a rural area. Rural training placements may help increase willingness to practice in rural areas, although a combination of strategies such as financial incentives and locating medical schools in rural areas is likely to have the greatest impact.^8^

We have previously argued that recruitment and retention efforts should not only focus on the job characteristics, organisation and professional training, but also on place, personal choices, and family needs.^9^ Families may be as much the target of community recruitment initiatives as practitioners themselves, and play an important independent role in maintaining community sustainability.^10,11^ The local community has been identified as an important factor in attracting and integrating a health care worker and their family.^12–15^ For example, research in Australia found that new doctors considering rural practice not only looked at the features of the job itself but also at factors such as childcare, schools and employment options for partners, as well the attractiveness of the natural environment.^16^ Linked issues are migration and sense of place, although research on health care recruitment and retention has not engaged with this place-based literature.^17,18^ A migration perspective shifts attention from the job to person, family and place, particularly with regard to retention.^19^

Rural communities are socially, culturally and economically distinct and initiatives to improve recruitment and retention will need to be tailored to local contexts, with a focus on an ‘engaged community’ and the need for a ‘whole of person’ and ‘whole of society’ approach.^1,19^ Research has considered community activism,^20^ including community-led action plans^21^ and community education and support,^22^ but there remains a gap in knowledge of what communities have tried and what has worked for them to help attract and integrate health care workers.

This study sought to contribute to closing this gap through exploring remote and rural community members’ experiences of trying to attract and retain health care staff and their families to their area in the National Health Service (NHS) in the United Kingdom (UK). It further aimed to understand how community initiatives were received by staff and families who have moved to work and live in a rural area, to assess which initiatives seem to have been more or less successful and why, and to provide resources for other communities and the NHS based on this learning.

## Methods

We used a qualitative design involving five case studies, three in Scotland and two in England. The study was co-designed with public research partners who were co-investigators and are co-authors of this paper. Our research team included members with backgrounds in health services research and medical sociology, human geography, medical education, clinical academics in primary and secondary care with experience of remote and rural practice, and health economists with expertise in workforce. We were supported by an external advisory panel and a wider lay panel of patient and public partners.

### Theoretical framing

The study was informed by an ‘asset-based community development’ (ABCD) approach,^23^ which seeks to understand the strengths, assets and resources within communities, which, while often unrecognised, can support the development of locally led solutions to community problems. Assets may be individual and collective and include place-based assets as well as institutions and social networks. Our approach was also informed by theories of place and migration studies,^12,17,18^ and we adopted a place-and-person based approach to understand health care workers’ career choices and to propose a shift in language from ‘recruitment and retention’ to ‘moving and staying’ to reflect these theoretical influences.^9^

### Data collection

Potential case studies were identified purposively through a combination of searching for media reports, snowballing through the research team’s professional networks, and suggestions from our advisory panel and patient and public involvement partners. A ‘community’ should be located in a remote or rural area, and should have been actively involved in leading efforts to recruit and/or retain health care workers. The sampling pool was small however; it proved more difficult than expected to recruit cases. Our final sample comprised remote coastal and inland locations and islands. Fieldwork took place between April 2022 and February 2023.

For each case, we identified a lead individual, who was contacted by the research team and asked to identify local community members who may have been involved in local recruitment and retention initiatives, and health care workers who have moved to work in the community. From these participants we used snowball sampling to recruit family members, other health care workers and local residents with an insight into our topic. The number of participants per site was determined by relevance and the nature of local action, rather than a target number per case, with a total of 22 participants taking part across the five sites.

We conducted in-depth interviews, inviting participants to share their experience of local recruitment and retention initiatives, including what activities were initiated, what worked or did not work, and the role of the local context. Fieldwork was conducted partly remotely during the COVID-19 pandemic and partly in person. Interviews were audio-recorded and transcribed verbatim by a professional transcriber. Most interviews lasted around 60 minutes.

### Analysis

The entire research team was involved iteratively in data analysis, reviewing and discussing interview transcripts at regular meetings to start identifying themes and forming a view of the ‘story’ behind each case. Interview extracts were also shared with our advisory and lay panels to gather multiple perspectives on the data and to ensure our interpretations were grounded in the reality of remote and rural life. We also included in the analysis an interview conducted for a previous study with a GP in one of our selected sites, who consented to secondary analysis. Transcripts were analysed thematically; we drew deductively on asset-based community development principles as well as theories of place and migration, and used inductive coding to develop a coding framework.^24^ For example, we coded for specific types of human and place-based asset mentioned by participants, as well as inductive items such as interpersonal relationships and conflict. AM led the development of the coding framework in discussion with LL and ZS and applied it to the data.

We also developed a detailed, holistic case description for each site, drawing on transcripts and on observational fieldnotes to capture the detail of what each community did and why, the assets they were able to bring to the process, and help interrogate commonalities and differences across cases.

## Ethics approval

Ethical approval was granted by University of Aberdeen School of Medicine Research Board, SERB/2021/10/2186. As our case studies were small and potentially identifiable, we present findings thematically rather than by case to preserve participants’ confidentiality.

## Results

We begin documenting our findings by describing communities’ actions and assets. We then report on two key interpretive themes that we identified from our data: ‘Recruitment: power, labour and responsibility' and ‘Retention and social navigation'.

### Actions and assets

Initiatives observed in our case study sites ranged from highly purposeful, intentional campaigns to something more emergent and informal. Recruitment was the usual focus, with retention initiatives being less obvious or deliberate. Box 1 summarises the categories and types of recruitment and retention activities that we identified.

**Box 1.** Categories and types of recruitment and retention activities used by communities.

Stakeholder campaign groups
Contributing to job adverts
Promoting the area as a good place to work
Making a video
Social media campaigns
Collaborating with neighbouring practices
Help with sourcing housing and practice buildings
Organising or contributing to pre-application visits
Sourcing job opportunities for partners
Social navigation and buddying – introducing new people to local activities, clubs
Informal social integration (being welcoming and cultivating friendships)

For example, one community set up an action group and mobilised local politicians.

*There was about sixty people that turned up and our local [Member of Parliament] chaired the meeting […] And then there was ten people that stood and said that they would help […] None of us really had a background in anything to do with the NHS - so we weren’t nurses or doctors or anything like that. But we were really strong community voices that I think people respected. [….] It now has a more positive voice rather than being an action group – where it sounds like we’re attacking the NHS. We’ve never actually done that. We just wanted the truth from them*. (CAWH-02)

The group collaborated with the local GP practice to make a video featuring local people stating that they needed a new doctor, along with practice staff talking about professional and personal aspects of life in the location and footage of landscape and outdoor activities.

Commonly, these kinds of initiatives were accompanied by a social media campaign. The hope was that people who were not already actively looking for a rural post might see it and be tempted to apply.

*I did this sign, so basically we put this sign up as if it was just like an everyday advert, looking for a lost dog or whatever – but looking for a GP. And we put the highlights of the job and we hand wrote it to make it look like it was nice and friendly, and then we thought if we put in beautiful photos, we’ve then got something new to release every week so we’ll share it and then everyone in the community will share it and then all their friends will share it, and the idea was that eventually the right person would see this. But what was really important was that the person that would see it wasn’t looking for it and that was our challenge. Because if the person who’d seen it was looking for it, then they would have already found it but the person that we were looking for didn’t know that they were looking for it.* (CAWH-03)

One community specifically targeted social media groups for the kind of activities they thought might attract someone to a rural area.

*Basically I went and searched [social media] groups where I thought there was a correlation between doctors and the type of people we could imagine wanting to live in [community]. So we basically targeted outward bound type people, we targeted sailors, walkers, ramblers […] I just went round as many of these groups on [social media] and posted a picture with a job ad and just said, ‘If you know anyone who might be interested in this, please forward it on to them’*. (CAWH-08)

However, this participant noted that the mechanism by which their campaign reached the target audience was primarily because it was picked up by broadcast news, rather than directly through their own social media activity.

In addressing recruitment challenges, community campaigns drew on a range of assets. This often included the asset of their natural environment, involving *‘idyllic areas, with some turquoise sea in the background or gorgeous mountains’* (CAWH-08), as well as featuring opportunities for an outdoor lifestyle, local schools, restaurants and shops. Communities also drew on local individuals with specific skills as an asset for delivering initiatives, such as people with backgrounds in marketing, design and advertising; photographers and film-makers; people with broadcast media connections; communications experts; and those with time and organisational skills to contribute.

Conversely, a lack of assets could pose significant challenges to community-led initiatives, most importantly lack of affordable (or any) housing, of employment opportunities for other family members, and distance to secondary schools for older children.

*Property is in short supply, things that become available tend to be out of the region of most nurses’ salaries and so the two things together, even when people become interested and start thinking about moving up here and do what we’ve done, they quite often get as far as looking at the properties that are available and saying, ‘The place that we want isn’t available, when it comes on the market it’s gone within twelve hours.’ […] They just feel that they can’t break into that housing market. And the rental market is very limited*. (CAWH-11)

### Recruitment: power, labour and responsibility

A common theme across case studies was tension with local NHS health organisations responsible for local service provision and recruiting health care staff. There was a perception among community members of inaction and that the NHS invested minimal effort or care being invested into recruitment.

*We set up a [community] Healthcare Group and we were trying to persuade NHS [organisation] to listen to us and do something about the situation. [….] They hardly paid any attention to us at all, we weren’t a recognised body of any sort. So [….] a bunch of us put our names forward for the community council and they did start paying more attention then. We were a recognised body. And so that helped matters. But our argument has always been with the hierarchy, not with the people on the ground trying to do their jobs in very difficult circumstances. [The staff] were stuck between a rock and hard place*. (CAWH-05)

Inaction was often suspected to be not just a lack of effort, but rather a way to reorganise service provision that would save the NHS money. There was a sense of frustration among study participants who felt that NHS decisions-makers had limited local knowledge to understand the implications of the loss of services for the health of local people or the wider sustainability of their community.

*And they [local NHS organisation] were trying to cut down the service and centralise everything and we resisted. And amazingly we got where we did. Even when [healthcare staff] were only coming over for the day, you lost continuity. […] They [healthcare staff] didn’t know the basics of the community, they’d know the one or two people that they were dealing with, but not everybody else. So if something came up, they wouldn’t have any background on that person that a resident would have. […] It wasn’t even efficient because people were having to, for example, stay overnight in hospital because there was nobody at home. And the lifeboat was getting called out, on a fairly regular basis, because if anything came up and somebody needed attention, there was nobody in place to say if it was serious or it could wait until the morning.* (CAWH-05)

*We get that a lot with [the council]: ‘You do it’. That’s not what we’re here for, that’s what you get paid for – we don’t get paid, we’re volunteers here. And we get that a lot with a lot of other things, highways, street cleaning [….] But with this, yes, communication was very lax from the [NHS organisation]. They were making decisions without consultation with anyone*. (CAWH 15 + 16; joint interview)

Other participants also described examples where disagreements led to responsibility being passed back to communities to ‘do it themselves’.

*By this time we had all these letters from all the other doctors and other health people saying [community] needed a doctor. So at the public meeting, I said that ‘What you’ve done is not good, it hasn’t been good enough.’ So [health official] said to me, or to us, ‘Well if you think you can do any better, you do it.’ … And I thought, ‘Oh my god, what have we done?’ Because that was pretty scary*. (CAWH-01)

This tension and sense of powerlessness was the catalyst for a range of community initiatives as described above. Some communities took ‘full control’ and ran a campaign, while others worked more collaboratively with the NHS. In one case, after the NHS had tried and failed to recruit by advertising several times in an ‘*old fashioned medical profession journal' *which *“almost has cobwebs”* (CAWH-08), the community council created its own social media campaign with little NHS involvement.

*I can’t really remember, I’m sure we would have spoken to them about it saying ‘Would you mind if we do this?’ I think it was about as much as that, I think it was just out of politeness. It was basically me and [another community member], who you’ve spoken to. [She] has got a background in marketing, I’ve got a background in marketing […] The two of us just saw the opportunity so we just ran with it. […] The authorities were aware of it. But having said that, I think we just ran with it, there weren’t any sign-offs or anything like that, we basically went and did it*. (CAWH-08)

In another case, two health care workers who collaborated with the community to take over a vacant GP practice had left, described the frustration of what they felt was a sham NHS application process.

Participant A: *The application process was outrageous, as far as I was concerned, because they [local NHS organisation] just didn’t want it to happen and I presume that was because of money. [….] If I’d not had a background of medical secretarial and PA work there’s no way we’d have succeeded with the application because they were making us jump through so many hoops.*

[…]

Participant B: *This particular practice seemed to have been run down and I got the feeling that they [NHS organisation] really, well, they all voted to close it […] The only reason that I could think of was because that they would save money by dispersing the list and less hassle. *(CAWH-21 & 22)

In rural and remote communities, individuals often fill several voluntary and paid roles. Getting involved in recruitment campaigns could generate significant work for these volunteers, and it was often the same people who appeared to step up. However, those involved tended not to think of what they did as work, or an intervention as such, but rather just what anyone would do as responsible local residents while acknowledging the effort and labour involved.

*My father used to say if you did anything good, constructive or helpful for other people, and he was thanked for it, he would say, ‘no, no, it’s just enlightened self-interest’, because he could see that in the future, it would be good if he’d done this. And I guess for all of us to have skilled and happy and contented health staff in our area, that’s enlightened self-interest. So I just feel if you put stuff into the social fabric of the area that you are in, it comes back to you and everybody else. *(CAWH-18)

It was only when prompted by a question about the work involved that this person acknowledged it could be *‘laborious’*.

*What it involved was persistence and going to meetings again and again and again and cultivating contacts and networks. So I guess that’s the work of it, it’s ‘do I want to go to this bloody meeting?’ That’s the laborious part of it, but if you are kind of gregarious, like me, then it’s also fun*. (CAWH-18)

Case study sites differed in the extent to which one or two individuals drove recruitment action, or whether it was a more distributed activity among several community members. The role of particular people could be variously interpreted, disputed, or resented, as well as praised. Thus, there were not only tensions and power imbalances between community and NHS organisations, but also within communities themselves, and dominant personalities could leave others feeling excluded.

*Everybody has got something to offer, that’s the way I look at it. [….] But obviously some people are more forefront, aren’t they, and I found that quite tricky sometimes because I tend to sit back if that happens and I just do my bit quietly […] So yes, I think when you’ve got somebody out-front that is very, very forceful, that’s tricky. [….] But at the same time, you’ve got somebody like that who wanted to lead the group and blah-de-blah and had all the energy. And that’s what you need as well. […] And without that kind of drive and energy, then things might never have happened. So you need it all, it’s just hard*. (CAWH-17)

### Retention and social navigation

In all our case studies, communities were successful in recruiting health care staff. Staff retention was however problematic in some cases, reflecting the more nuanced, informal and emergent nature of retention compared to formal recruitment initiatives. Our analysis highlighted the value of social navigation: actions by individuals or the community as a whole that help make people feel welcome and create a sense of belonging. Inevitably, in some cases the reasons for healthcare staff leaving were beyond the control of the community, often for complex social and personal reasons.

Wife*: It was a pull rather than a push that really took us away. And the things that were tricky, like the schooling etc, I think we would have managed them somehow or other, we would have adapted and managed them if it hadn’t had been that we were getting a pull [elsewhere] for family reasons […] I can’t think of anything else the community could have done that would have made any difference*. (CAWH-09 & 10)

Nonetheless the importance of the community being ‘*really friendly, really welcoming*' (CAWH-13) was often stressed, even before health care workers arrived or had accepted a post. We saw examples of community members being on interview panels, meeting applicants to tell them about the area, or inviting them to community events before they formally took up post. In some cases community members also helped people look for housing or advised on possible job opportunities for partners. This, in turn, could lead to those having experienced such a welcome themselves to do the same for new arrivals.

*[My husband] came here for his interview without me, because I’d just had a baby […] The hotel was at that point run by a couple who had a small baby who was a little bit older than [our daughter], so literally from the second [my husband] got in they were obviously, they were wanting to encourage, they were desperate for a doctor. There was a couple of other candidates as well but…so the lady who was running the hotel immediately, [my husband] was messaging me saying ‘I’ve found you a friend, she says you can ring her any time if you want to talk about breastfeeding or whatever’, all this kind of thing.*

[….]

*We came to visit and you know, I was just immediately grabbed by this group of women who had small children. We went to have a little look at the house, another friend was banging on the door, we were looking at the doctor’s house for the surgery, she sort of popped over, she was like, ‘Hi, I’m [name], this is my baby, please come to toddler group on Friday, come and meet everybody’. [….] I’ve tried to do the same when people have moved here*. (CAWH-19)

Others also described an overt and intentional approach to helping new arrivals to feel welcome and integrated, perhaps by arranging coffee and suppers or inviting people to get involved in volunteering or leisure activities. Commonly this was led by particular individuals, sometimes with retention specifically in mind, sometimes just in order to be kind and build the community. However, reliance on individuals acting as social navigator may also be problematic.

*He is great, but you can’t just rely on one person. We need to form a welcoming committee and make sure that when these people come to work here, they are absorbed into the social network of the village quickly….. almost sort of when you offer someone a job, you say ‘here’s the accommodation that you’ll be able to afford and here is your social passport…’ [laughing] ‘When you arrive, here is your list of options, tick the ones you are interested in and we will facilitate that for you’, so when you do arrive you feel completely – I’ve got this menu of options, I’m appreciated, I’m going to be able to have some social time and not feel isolated and I’ve got this place to live. Perfect*. (CAWH-11)

By contrast, in other communities it seemed that retention was given little consideration and all their energy had been focused on recruitment. Questions about what the community had done to retain newly recruited GPs were sometimes met with puzzlement, as if this was not something they felt the community could contribute to. One participant talked about what a missed opportunity it had been not to take the new healthcare worker round the community to introduce them to some of the people who had been involved in the recruitment campaign, and make them feel welcome. This healthcare worker left a few years later, although this may have been for many other reasons.

The kind of informal and often unconscious social navigation work which can encourage healthcare staff to stay often remains undocumented and under-recognised, even in those places which seem to do it well. Our case studies were dominated by narratives of recruitment, and reflections on retention often emerged only after careful prompting.

## Discussion

This study found that remote and rural communities can play a valuable and effective role in recruitment and retention of health care staff. Communities in our study drew on a range of local assets to help attract and retain health care staff and their families. Many of these assets were place-related. Showcasing these assets requires not just good campaign design but also the ability to get the campaign in front of potential candidates. Here local people with marketing and design skills and good social networks may be another useful asset. Where the NHS is struggling to recruit, community members could be invited to help create or contribute to recruitment drawing on their local knowledge of what attracts people to that area. This study provides UK-specific evidence to support international research on the role that community engagement and activism can play.^20–22^ As Urquhart^25^ suggests, communities have important local knowledge about what attracts people to their area to inform successful interventions, and their involvement can encourage a sense of ownership for helping to integrate new health care staff and their families.

Our findings add to international evidence that what attracts people is not merely the salary or the role itself, important as these are, but a combination of personal and family preferences and needs, influenced by life stage and preferences, and the attraction (or not) of a particular place. Elsewhere we have argued that reconceptualising recruitment and retention as ‘moving and staying’ better captures this more holistic understanding of people’s motivation to take up a post in remote or rural areas, and what keeps them in place.^9^ This has been referred elsewhere to ‘Attract-Connect-Stay’^26^ or as ‘coming here’, ‘being here’, and ‘staying here’.^27^

Staying here, retention, is a neglected area. Even where recruitment interventions are successful, their impact may be short-lived. In this study we identified case studies primarily because of high profile recruitment campaigns; we had to dig deep to explore more hidden, socially enacted retention actions. These are poorly documented and often not recognised, even by those involved. Describing this ‘social navigation’ work, both intentional and unintentional, to create a sense of belonging is a key contribution of this study. As Mandal and Phillips’ work on physician retention in rural Canada suggests, that sense of belonging “is built via bilateral active efforts at community engagement, reciprocity, social integration of family and workplace collegiality” (p.1).^17^ The formation of community stakeholder groups can help lessen the burden on individuals.

We used the asset-based approach, which has been a useful analytic lens to understand community-led recruitment and retention. However, it has important limitations. Rather than empowering the community, it is argued that it risks placing the whole burden of maintaining and improving their area on individuals and communities, diverting attention from wider infrastructure and funding issues which are beyond the control of the community, and enabling central authority to abrogate responsibility.^28^ The absence or poor quality of assets such as housing, transport links, Internet connectivity, employment opportunities for other family members and schooling can undermine community campaigns. Asset-based approaches may worsen inequalities in and between already under-served areas, and some communities will be less able than others to take this kind of action.

The community members we spoke to usually got involved because they felt they had to, and sometimes described a degree of weariness at having to repeatedly fight for the viability of their community. Rural and remote places are often romanticised and their residents stereotyped as ‘resilient’.^29^ But the discourse of resilience brings its own problems and burden. ‘Resourcefulness’ has been proposed as an alternative term to ‘resilience’, reflecting a more bottom-up approach focused on social justice and challenging power relations.^30^

This is not to say that communities cannot take useful action and exercise some control over their destiny, but rather that they cannot be expected to solve all recruitment and retention problems, which still require government and health organisation action.

What works in one community may not be appropriate or possible in another context, and there is a risk that if every community produces a video, for example, the novelty and profile of such campaigns will be diluted. However, what this study offers is a range of ideas for other communities to explore. In order to support other communities, we have created an online ‘library of examples’,^31^ which will be disseminated publicly through the new Scottish National Centre for Remote and Rural Health and Care, part of NHS Education for Scotland.

### Study limitations

This UK-based study explored in detail community-led initiatives to support health care recruitment and retention in rural and remote areas. It provides novel exploratory evidence on an under-researched topic, and contributes to the limited international evidence base. However, this was a relatively small study. Recruitment of case studies, and of participants in some sites, was challenging, and we had to reduce our planned number of case studies. We had hoped to conduct place-based fieldwork in the communities studied, but this was not possible as data collection coincided with the COVID-19 pandemic in the UK.

Demonstrating causality is not in the aim of an exploratory study of this kind. However, the data we were able to collect offered rich, detailed and wide-ranging perspectives that enabled us to move beyond a narrow job focus to a whole person, whole family and whole society perspective.

## Conclusion

Successful recruitment and retention needs to focus widely, not just on the whole person but on their families, their collective preferences and needs, and social integration to the community. Our findings suggest that there is an important role for communities to play in recruitment and retention, but they cannot be expected to solve all the issues. Central and regional government and the NHS could engage in a more agile, tailored and nuanced way with communities at an earlier stage, benefiting from their local contextual knowledge and energy to develop place specific solutions and approaches that will encourage health care staff to move to, and ultimately stay in, a rural community.
